# Data linkage and statistical modelling to provide stratified risk assessment for HAI

**DOI:** 10.23889/ijpds.v1i1.297

**Published:** 2017-04-19

**Authors:** Kim Kavanagh, Jiafeng Pan, Chris Robertson, Marion Bennie, Charis Marwick, Colin McCowan

**Affiliations:** University of Strathclyde; NHS National Services Scotland; University of Dundee; University of Glasgow

## Objectives

The use of "real-time" data to support individual patient management and outcome assessment requires the development of risk assessment models. This could be delivered through a learning health system by the building robust statistical analysis tools onto the existing linked data held by NHS Scotland’s Infection Intelligence Platform (IIP) and developed within the Scottish Healthcare Associated Infection Prevention Institute (SHAIPI).

This project will create prediction models for the risk of acquiring a healthcare associated infection (HAI), and particular outcomes, at the point of GP consultation/ hospital admission which could aid clinical decision making.

## Approach

We demonstrate the capability using the HAI Clostridium difficile (CDI) from 2010-2013. Using linked national individual level data on community prescribing, hospitalisations, infections and death records we extracted all cases of CDI and by comparing to matched population-based controls, examined the impact of prior hospital admissions, care home residence, comorbidities, exposure to gastric acid suppressive drugs and antibiotic exposure, defined as both cumulative (total defined daily dose (DDD)) and temporal antimicrobial exposure in the previous 6 months, to the risk of CDI acquisition. Antimicrobial exposure was considered for all drugs and the higher risk broad spectrum antibiotics (4Cs). Associations are assessed using conditional logistic regression. Using cross-validation we assess the ability of the model to accurately predict CDI infection. Risk scores for acquisition of CDI are estimated by combining these predictions with age and gender population incidence.

## Results

In the period 2010-2013 there were 1446 cases of CDI with matched 7964 controls. A significant dose-response relationship for exposure to any antimicrobial (1-7 DDDs OR=2.3 rising to OR=4.4 for 29+ DDDs) and, with elevated risk, to the 4C group (1-7 DDDs OR=3.8 rising to OR=17.9 for 29+ DDDs). Exposure elevates CDI risk most in the month after prescription but for 4C antimicrobials the elevated risk remains 6 months later (4C OR=12.4 within 1 month, OR=2.6 4-6 months later). The risk of CDI was also increased with more co-morbidities, previous hospitalisations, care home residency, increased number of prescriptions, and gastric acid suppression. 

## Conclusion

Despite limitations to current application in practice,(paucity of patient level in-hospital prescribing data and constraints of the timeliness of the data), when fully developed this system will enable risk classification to identify patients most at risk of HAI and adverse outcomes to aid clinical decision making.

